# Prevalence and predictors of medication non-adherence in children with inflammatory bowel disease in China: A cross-sectional study

**DOI:** 10.3389/fphar.2022.1062728

**Published:** 2022-12-01

**Authors:** Yuanyuan Wu, Lingfei Huang, Jin Sun, Huijuan Wang, Luo Fang, Jing Miao

**Affiliations:** ^1^ Department of Pharmacy, The Children’s Hospital, Zhejiang University School of Medicine, National Clinical Research Center for Child Health, Hangzhou, China; ^2^ Department of Pharmacy, The Cancer Hospital of the University of Chinese Academy of Sciences (Zhejiang Cancer Hospital), Institute of Basic Medicine and Cancer (IBMC), Chinese Academy of Sciences, Hangzhou, China; ^3^ Research Center for Clinical Pharmacy, Zhejiang University, Hangzhou, China

**Keywords:** inflammatory bowel disease, children, medication adherence, predictors, risk factors

## Abstract

**Background:** Non-adherence to physician-prescribed medications, especially oral medications, is common in children with inflammatory bowel disease (IBD), and medication non-adherence is associated with poorer outcomes in IBD. Therefore, understanding and improving medication adherence in children with IBD is critical for optimizing treatment and improving treatment outcomes. Despite the relatively high prevalence of IBD in children in China, to date, very little is known about medication adherence in these patients.

**Objective:** The aim of this study was to investigate the prevalence of medication non-adherence and its risk factors in children with IBD in China to provide a basis for developing adherence improvement strategies.

**Methods:** A cross-sectional design was employed. Children (aged <18 years) with IBD who visited the Children’s Hospital, Zhejiang University School of Medicine, from September 2020 to December 2021 were included. Data were collected by a general information questionnaire, the 4-item Medication Adherence Report Scale (MARS-4) and Crohn’s and Colitis Knowledge (CCKNOW) questionnaire. Subsequently, forward stepwise binary logistic regression analysis was performed to determine independent predictors of medication non-adherence.

**Results:** A total of 119children were included in this study. The results showed that 33 (27.73%) and 86 (72.27%) children had poor and good medication adherence, respectively. Of these, 83 (69.75%) had forgotten to take their medications sometimes, often, or always. On binary logistic regression, we found that the incidence of medication non-adherence in children with IBD course of 3 years and above [*OR* 4.82 (95%*CI*: 1.47-15.88); *p* = 0.01] was significantly higher than that in children with course of 3 months to 1 year, whereas children with higher parental CCKNOW scores [*OR* 0.77 (95%*CI*: 0.67-0.88); *p* = 0.00] had significantly lower incidence of medication non-adherence than those with lower parental CCKNOW scores, and the results of the correlation between parental knowledge scores of the four categories and children’s medication adherence showed that drug knowledge scores (*r* = 0.36, *p* < 0.00) and complication knowledge scores (*r* = 0.24, *p* = 0.01) were positively correlated with medication adherence.

**Conclusion:** Poor medication adherence in children with IBD in China was common, and forgetting to take medication was the main barrier. Longer disease duration (3 years and above) in children could act as a risk factor for medication adherence, whereas higher level of parental knowledge about IBD could act as a protective factor, and one interesting novel finding was that the level of parental knowledge about drug and complication were significantly correlated with medication adherence in children with IBD. Our results may inform on the design and implementation of medication adherence interventions for children with IBD.

## Introduction

Inflammatory bowel diseases (IBD), including Crohn’s disease (CD) and ulcerative colitis (UC), are chronic inflammatory diseases of the gastrointestinal tract. Approximately 25% of patients first develop symptoms under 20 years of age, while only 4%–10% are diagnosed before the age of 6 years (Rosen et al., 2015; Afzali and Katz, 2018). The incidence of pediatric IBD has risen rapidly in recent years, and it has emerged as a global disease in developed and developing nations (Benchimol et al., 2011; Benchimol et al., 2017; Piovani et al., 2020). Symptoms of IBD include gastrointestinal symptoms such as abdominal pain, diarrhea, and blood in stool, and non-gastrointestinal symptoms such as arthritis and autoimmune liver disease, which lead to slow growth, delayed puberty, and decreased quality of life. In addition, psychiatric comorbidities, particularly depression and anxiety, are common in pediatric IBD patients (Loftus et al., 2011; Keerthy et al., 2016).

The treatment goals of IBD are to induce and maintain clinical remission, achieve mucosal healing, and reduce the need for surgeries and hospitalizations (Xiao et al., 2014). Medications, including corticosteroids, 5-aminosalicylates, immunomodulators, and biological agents, are the primary means to achieve these treatment goals. However, non-adherence to physician-prescribed medications is common in children with IBD (Hommel et al., 2009; Spekhorst et al., 2016). Previous studies have shown that poor adherence to medication can lead to disease recurrence or progression, resulting in the need to step-up therapy, reducing health-related quality of life, and increasing treatment costs (Gray et al., 2012; LeLeiko et al., 2013; Pearce and Fleming, 2018; Plevinsky et al., 2019; Collyer et al., 2020). Therefore, understanding and improving medication adherence in children with IBD is critical for optimizing treatment and improving treatment outcomes. However, very little is known on this subject in China.

Oral medications are a mainstay of IBD treatment, non-adherence to oral medications is regarded as a pervasive problem in the management of IBD (Greenley et al., 2012). Therefore, we focus our discussion and analysis on the oral medication adherence in children with IBD. The first step in understanding adherence is quantifying its incidence. This can be performed by self-report, objective measures, or mixed indicators; the most commonly used is the patient-self-reported medication adherence scale because of its effectiveness, reliability, convenience, and cost-effectiveness. However, there are literatures showing that subjectively reported adherence (e.g., self-report, parent-report) can lead to overestimates of medication adherence due to recall bias and social desirability (Wu et al., 2013; Spekhorst et al., 2016). Then, some scholars have found that subjectively and objectively reported adherence were consistent and correlated (Wu et al., 2013; Selinger et al., 2019). Selinger et al. (2019) suggested the Medication Adherence Report Scale (MARS) and Visual Analog Scales (VAS) could be used as simple and effective screening tools for medication non-adherence in patients with IBD in clinical practice. The 4-item Medication Adherence Report Scale (MARS-4) is a widely used adherence-specific self-reported questionnaire that has been translated into several languages and has been shown to have good reliability and validity in assessing medication adherence in patients with different diseases (Horne et al., 2009; Jacobsen et al., 2009; Jeganathan et al., 2017). However, a Chinese version of the MARS-4 is not currently available. Therefore, in this study, we sought to translate the English version of the MARS-4 into Chinese, evaluate its psychometric properties, and explore the predictors of non-adherence to anti-inflammatory bowel disease medication in a group of children with IBD in China.

## Methods

### Study design and patients

This cross-sectional study was conducted at the Children’s Hospital of Zhejiang University School of Medicine. We enrolled children (age <18 years) who visited the hospital between September 2020 and September 2021 and who had been diagnosed with IBD for at least 3 months and taking oral IBD medicines in the prior 3 months.

This study was approved by the Ethics Committee of the Children’s Hospital, Zhejiang University School of Medicine (2020-IRB-133). Written informed consents were obtained from the children’s parents or guardians, and children over 8 years old provided written assent.

### Data collection

Two trained pharmacists approached patients who met the inclusion criteria and explained the purpose, procedure, risks, and benefits of the study to them and their parents. If the children (≥8 years old) and their parents agreed to participate, they were asked to complete questionnaires that included baseline demographic family and clinical information. Parental disease-related knowledge level was evaluated by the validated Chinese version of Crohn’s and Colitis Knowledge (CCKNOW) questionnaire, and children’s medicine adherence was assessed using the MARS-4. The CCKNOW questionnaire was completed by child’s parent, while the MARS-4 was completed by the child (child aged ≥12 years) or by his/her parent (child aged <12 years), as the MARS questionnaire has a Flesch-Kincaid Readability Grade Level of 6.0 and is considered appropriate only for subjects aged ≥12 years (Jeganathan et al., 2017).

In addition, the same pharmacist re-surveyed a randomly selected subset of 30 patients by telephone interview within 4 weeks to assess the test-retest reliability of MARS-4. Patients for the re-interview were selected by generating a random sample from all enrolled patients using SPSS 24.0.

### Medication adherence report scale

Adherence to medications was assessed using the MARS-4, which has been validated in a range of patients, including those with IBD (Horne et al., 2009; Selinger et al., 2013; Tiao et al., 2017). The four items are scored on a 5-point Likert scale, ranging from always (1) to never (5), and summed to yield a total score between 4 and 20, with higher scores indicating higher levels of adherence. Participants scoring ≤16 were classified as having low adherence, and those scoring ≥17 were classified as high adherence (Horne et al., 2009).

### Parental knowledge about IBD

A linguistically and culturally validated translation of the CCKNOW questionnaire was used to assess parental knowledge about IBD (Zhu et al., 2012). The CCKNOW is a self-administered IBD knowledge questionnaire which was developed by Eaden et al., in 1999 (Eaden et al., 1999). Consists of 24-item covering four areas of IBD management: 1) general knowledge, including anatomy, 2) medication, 3) diet, and 4) complications (Colombara et al., 2015).

### Statistical analysis

All raw data were double-entered into Microsoft Excel for Office 365 MSO by dedicated study staff, and then exported to SPSS (version 24.0) for analysis. Descriptive statistics were used to assess the participants’ sociodemographic and medical characteristics. Normally distributed continuous data were expressed as the mean (range) and non-normally distributed continuous data as the median (IQR), while categorical variables were expressed as a percentage.

The internal consistency reliability of the MARS-4 was estimated by calculating the Cronbach’s alpha coefficient, where a value of 0.70 or higher indicates good internal consistency. Test-retest reliability was analyzed after a 4‐weeks interval with an intraclass correlation coefficient (ICC) value > 0.70 indicating acceptable reliability (Bland and Altman, 1986). Construct validity was identified by an exploratory factor analysis (EFA) with principal components.

For group comparison, 2-tailed unpaired t-test or Mann-Whitney U-test was used for continuous variables, and Chi-square test for categorical variables. Variables that were statistically significant or had a trend toward significance (*p* < 0.2) or were of *a priori* clinical significance were included in a multivariable analysis with forward stepwise ordinal logistic regression, to evaluate independent factors associated with medication non-adherence. Odds ratios (ORs) and 95% CIs were used to measure the effect of predictors. All results are presented as two-tailed values with statistical significance for *p* values < 0.05.

## Results

### Demographic and medical characteristics

A total of 119 participants, with a median age of 12.58 (8.58, 14.00) years, were recruited. Among them, 80 (67.23%) were male, 95 (79.83%) were diagnosed with CD, and 14 (11.76%) were diagnosed with UC. The participants’ characteristics are presented in Table 1.

**TABLE 1:** Demographics and clinical characteristics of the study participants (*n* = 119).

	Adherence (*n* = 86)	Non-adherence (*n* = 33)	*χ* ^*2*^	*P*
Age, *M (IQR)*, yr	12.67 (9.65–13.92)	12.58 (8.17–15.71)	−0.59^a^	0.56
Gender, *n* (%)			1.51	0.22
Male	55 (63.95)	25 (75.76)		
Female	31 (36.05)	8 (24.24)		
Diagnosis, *n* (%)			0.85	0.65
CD	70 (81.40)	25 (75.76)		
UC	10 (11.63)	4 (12.12)		
VEO-IBD	6 (6.98)	4 (12.12)		
IBD duration, *n* (%)			6.19	0.05
3 months-1 year	40 (46.51)	10 (30.30)		
1–3 years	34 (39.53)	12 (36.36)		
3 years and above	12 (13.95)	11 (33.33)		
Disease severity, *n* (%)			5.43	0.14
Remission	52 (60.47)	22 (66.67)		
Mild	19 (22.09)	10 (30.30)		
Moderate	12 (13.95)	0 (0.00)		
Severe	3 (3.49)	1 (3.03)		
No. Of IBD medications, *n* (%)			0.05	0.82
1 medication	81 (94.19)	30 (90.91)		
2 or more medications	5 (5.81)	3 (9.09)		
Medication frequency, *n* (%)			0.20	0.66
Once a day or less	70 (81.40)	28 (84.85)		
Twice a day or more	16 (18.60)	5 (15.15)		
Health insurance, *n* (%)			0.29	0.87
No health insurance	20 (23.26)	9 (27.27)		
Rural new cooperative medical scheme	38 (44.19)	13 (39.39)		
Urban resident basic medical insurance	28 (32.56)	11 (33.33)		
Average monthly income, *n* (%)			0.10	0.61
Less than RMB 2500	22 (25.58)	10 (30.30)		
RMB 2500–5000	38 (44.19)	16 (48.48)		
More than RMB 5000	26 (30.23)	7 (21.21)		
Parental CCKNOW score, *M (IQR)*	9.00 (6.75–11.00)	5.00 (3.50–8.00)	−4.04^a^	0.00

CD, Crohn disease; UC, Ulcerative Colitis.

VEO-IBD: very early onset inflammatory bowel disease; IBD: inflammatory bowel disease.

CCKNOW: Crohn’s and Colitis Knowledge questionnaire.

^a^ Mann-Whitney test.

### Psychometric evaluation

#### MARS-4 reliability

The MARS-4 showed good internal consistency, with a Cronbach’s alpha coefficient of 0.75. When deleting one item at a time, we found a Cronbach’s alpha that varied between 0.60–0.76 (Table 2). To assess test-retest reliability testing, 30 questionnaires were completed and returned. We found excellent agreement between the test and retest, with an ICC of 0.73 (95% *CI* 0.51-0.86).

**TABLE 2:** Reliability test of MARS-4 (*n* = 119).

MARS-4 item	CITC	Cronbach’s ⍺ if item deleted	Cronbach’s ⍺
1. I forget to (give my child to) take these IBD medicines	0.54	0.70	0.75
2. I alter the dose of these IBD medicines (of my child)	0.41	0.76	
3. I stop (my child) taking these IBD medicines for a while	0.60	0.64	
4. I decide to (let my child) miss out on a dose	0.67	0.60	

MARS, Medication adherence report scale; CITC, Corrected Item—Total correlation.

#### MARS-4 validity

The construct validity of the MARS-4 was evaluated using EFA. The Kaiser-Meyer-Olkin measure of sampling adequacy (KMO = 0.72) and Bartlett’s test of sphericity (*χ2* = 118.35, *df* = 6, *p* < 0.00) indicated that the data were appropriate for factor analysis. The results of the factor analysis revealed a single factor that accounted for 57.70% of the variance, which indicated good construct validity as they clearly measured one construct i.e. non-adherence.

### Medication adherence

**FIGURE 1:**
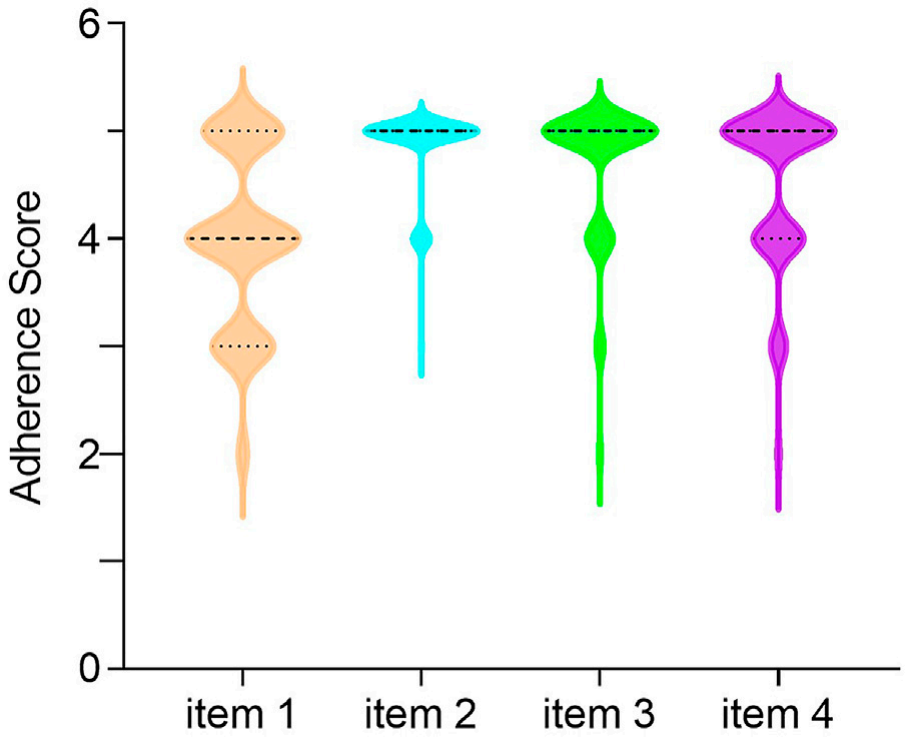
The distributions of scores for each item of MARS-4.

The mean (SD) medication adherence score of 119 children with IBD was 18.04 (1.99), 33 (27.73%) and 86 (72.27%) had low and high adherence, respectively. The distributions of scores for each item of MARS-4 are summarized in Figure 1. Unintentional non-adherence was evident with 83 (69.75%) of participants having forgotten to take IBD medications sometimes, often, or always. Intentional non-adherence was also common, with 45 (37.82%) of the participants reporting that they decided to miss doses, while 29 (24.37%) reported that they had stopped taking their IBD medications sometimes, often, or always. However, only 16 (13.45%) participants reported that they altered the dose of IBD medications. In addition, there were 68 children (≥12 years old) completed the MARS-4 while 51 parents completed it. Parent reported non-adherence (31.37%) was higher than child self-reported non-adherence (25.00%), the difference being not statistically significant. Unintentional non-adherence was evident, with a parent-reported score of 4.06 (0.93), higher than a child-reported score of 3.96 (0.74), but there was no statistical significant difference between them. And for intention non-adherence, including items 2 to 4, child-reported scores tended to be higher than parent-reported scores, but again, the differences were not statistically significant. Furthermore, there was no statistical difference between child-reported total score of 18.07 (1.74) and parent-reported total score of 18.00 (2.29) (Table 3).

**TABLE 3:** The scores for the MARS questionnaires completed by participating parents and children.

Measure	N	Score mean (SD)					Number (%) of non-adherence
		Item 1	Item 2	Item 3	Item 4	Total	
MARS-4 (completed by parent)	51	4.06 (0.93)	4.80 (0.45)	4.65 (0.72)	4.49 (0.76)	18.00 (2.29)	16 (31.37)
MARS-4 (completed by child)	68	3.96 (0.74)	4.90 (0.31)	4.69 (0.61)	4.51 (0.70)	18.07 (1.74)	17 (25.00)

MARS, Medication adherence report scale.

### Parental knowledge about IBD

CCKNOW scores ranged from 0 to 24; higher scores indicated higher level of knowledge about IBD of the parents. Table 4 shows the correct answer rate for the overall level of CCKNOW and for each item and category. The lowest correct answer rate was in the category of drug knowledge (28.24%), followed by complication knowledge (30.81%), general knowledge (36.13%), and the highest in diet knowledge (46.64%), while the overall correct answer rate was 34.03%. The total score of the CCKNOW was 8.00 (5.00-10.00). Table 5 shows the results of the correlation between parental knowledge scores of the four categories and children’s medication adherence, drug knowledge scores (r = 0.36, *p* < 0.00) and complication knowledge scores (*r* = 0.24, *p* = 0.01) were positively correlated with medication adherence.

**TABLE 4:** The correct answer rate for the overall level of CCKNOW and for each item and category.

The item and category of CCKNOW	Parents who answered correctly (%)
General knowledge (11 questions)	473^c^ (person-time) (36.13)^a^
Item 3: Proctitis: is a form of colitis that affects the rectum or back passage only	40 (33.61)
Item 4: Patients with inflammatory bowel disease are probably cured if they have been symptom free for 3 years: False	79 (66.39)
Item 5: Inflammatory bowel disease runs in families: True	21 (17.65)
Item 8: The terminal ileum: is a section of the bowel just before the large intestine	34 (28.57)
Item 9: During a flare up of inflammatory bowel disease: The platelet count in the blood rises	28 (23.53)
Item 15: Which one of the following statements is false? Ulcerative colitis is least common in Europeans and North Americans	56 (47.06)
Item 17: The length of the small bowel is approximately: 20 feet	31 (26.05)
Item 18: The function of the large bowel is to absorb: water	44 (36.97)
Item 19: Another name for an ileorectal anastomosis operation with formation of a reservoir is: pouch	8 (6.72)
Item 22: There are millions of tiny “hairs” in the small bowel to increase the absorptive surface. They are called: Villi	47 (39.50)
Item 23: Which one of the following is not a common symptom of inflammatory bowel disease? Headache	85 (71.43)
Diet knowledge (2 questions)	111 (person-time) (46.64)^a^
Item 1: People with inflammatory bowel disease are not allowed to eat dairy products: False	72 (60.50)
Item 2: Elemental feeds are sometimes used to treat Crohn’s disease and ulcerative colitis. They: are very easy to digest	39 (32.77)
Drug knowledge (5 questions)	168 (person-time) (28.24)^a^
Item 10: Steroids (such as prednisolone/prednisone/budesonide/hydrocortisone): can be given in the form of an enema into the back passage	14 (11.76)
Item 11: Immunosuppressive drugs are given to inflammatory bowel disease patients to: reduce inflammation in the bowel	74 (62.18)
Item 12: Sulfasalazine: can be used to reduce the frequency of flare ups	11 (9.24)
Item 13: An example of an immunosuppresive drug used in inflammatory bowel disease is: Azathioprine	54 (45.38)
Item 16: Male patients who take sulphasalazine: have reduced fertility levels that are reversible	15 (12.61)
Complication knowledge (6 questions)	220 (person-time) (30.81)^a^
Item 6:Patients with inflammatory bowel disease can get inflammation in other parts of the body as well as the bowel: True	66 (55.46)
Item 7:A fistula: is an abnormal track between 2 pieces of bowel or between the bowel and skin	29 (24.37)
Item 14: If a woman has Crohn’s disease: her pregnancy will always have complications	10 (8.40)
Item 20: If a part of the bowel called the terminal ileum is removed during surgery the patient will have impaired absorption of: Vitamin B12	9 (7.56)
Item 21: Patients with IBD need to be screened for cancer of the colon. Which one of the following statements about screening is true? Screening should be offered to all patients with ulcerative colitis which has lasted for 8–10 years	19 (15.97)
Item 24: If a child has inflammatory bowel disease; he/she probably will not: be as tall as his or her friends	87 (73.11)
Total	972 (person-time) (34.03)^b^

^a^ The correct answer rate of knowledge in this category.

^b^ The correct answer rate of knowledge in all question items.

^c^ Person-time, One person answered correctly for one time was defined as one person-time.

CCKNOW, The Crohn’s and Colitis Knowledge questionnaire; IBD, Inflammatory bowel disease.

**TABLE 5:** Correlation between medication adherence and parental CCKNOW scores of children with IBD (*n* = 119).

	General knowledge	Diet knowledge	Drug knowledge	Complication knowledge	Total score of CCKNOW
Medication adherence	0.11	0.03	0.36^a^	0.24^a^	0.26^a^

CCKNOW, Crohn’s and Colitis Knowledge questionnaire.

^a^ *p* values < 0.01 were considered statistically significant (two-tailed).

### Predictors of medication non-adherence

Compared with participants with high medication adherence (*n* = 86), those with non-adherence (*n* = 33) had longer disease duration (*p* < 0.05), and their parents had lower CCKNOW scores (*p* < 0.01) (Table 1). When adjusted for variables that were significant in univariate analysis as well as other variables defined *a priori* (age, gender, disease severity: remission *vs*. flare, number of IBD medications, medication frequency and economic level of family), we found that disease duration and parents’ CCKNOW scores were also independent predictors of medication non-adherence in children with IBD. The incidence of medication non-adherence in children with IBD course of 3 years and above [*OR* 4.82 (95% *CI*: 1.47-15.88); *p* = 0.01] was significantly higher than that in children with course of 3 months to 1 year. Furthermore, children with higher parental CCKNOW scores [*OR* 0.767 (95% *CI*: 0.67-0.88); *p* = 0.00] had significantly lower incidence of medication non-adherence than those with lower parental CCKNOW scores. (Table 6).

**TABLE 6:** Independent predictors for medication non-adherence in children with IBD.

Predictors	*B*	*S. E*	*Wald χ* ^*2*^	*P*	*OR* (95%*CI*)
Disease duration					
3 months −1 year	NA	NA	NA	NA	1
1–3 years	0.28	0.52	0.29	0.59	1.33 (0.48–3.71)
3 years and above	1.57	0.61	6.70	0.01	4.82 (1.47–15.88)
Parents’ CCKNOW score	−0.27	0.07	13.79	0.00	0.77 (0.67–0.88)

The reference category for disease duration was the first tertile (3 months −1 year).

IBD: Inflammatory Bowel Disease.

CCKNOW, Crohn’s and Colitis Knowledge questionnaire.

NA, Not applicable.

## Discussion

IBD is a chronic disease requiring lifelong treatment regimens to maintain remission. Unfortunately, poor adherence to oral medication is quite prevalent. Objective studies reveal medication non-adherence rates up to 93% in pediatric patients with IBD (Oliva-Hemker et al., 2007; Hommel et al., 2009; Ingerski et al., 2010; Spekhorst et al., 2016). The assessment and promotion of oral medication adherence is very complex. MARS could be used as a simple and effective screening tools for medication non-adherence in patients with IBD in clinical practice (Selinger et al., 2019). To our knowledge, this study is the first to demonstrate the reliability and validity of the Chinese version of the MARS-4 in assessing medication adherence in Chinese children with IBD. The study shows that the internal consistency reliability, test-retest reliability, and structural validity of the scale are relatively good, indicating that it can be used to measure medication adherence in Chinese children with IBD. However, it is worth mentioning that Item 2 has an item-total correlation below 0.5, this result is consistent with that of a previous study (Jacobsen et al., 2009). The reason for this may be that fewer patients changed the dose. Further, patients had access to a WeChat group with their children’s doctors, allowing them to get clear and timely answers when they had any questions regarding the medication use of their children at home, rather than arbitrarily changing the drug dosage themselves. This may be the reason why item 2 has a lower item-total correlation. Despite this, when this item was deleted, the Cronbach’s ⍺ only has a very small improvement with 0.006, and the internal consistency reliability of the Chinese version of the MARS-4 remains good. Based on this, MARS-4 was used in this study to assess children’s medication adherence. We found that nearly 70% of them forgot to take medication, while few changed the medication dosage, which were similar to the findings of (Ingerski et al., 2010), (Schurman et al., 2011). The most commonly reported medication adherence barrier was forgetfulness, for which some scholars have adopted a variety of reminder systems, both traditional, such as pill boxes, and novel, such as email and telephone alerts, have been considered as useful behavioral interventions for unintentional non-adherence (Bermejo et al., 2010). Therefore, in future research, we can adopt medication reminder systems, which may be able to promote children’s medication adherence.

This is the first study to investigate medication adherence and analyze the predictors of non-adherence to medication in Chinese children with IBD. Our results showed that about a quarter of children with IBD have poor medication adherence, which is higher than the rate in the United States (14.61%–17.47%) (Greenley et al., 2015), but similar to that in South Korea (31.2%) (Lim et al., 2020), lower than that in Jordan and the United Kingdom (36.2%) (Alsous et al., 2020), and much lower than that in Japan (43.0%) (Tanaka et al., 2021). Non-adherence to medication in children with IBD is common worldwide. To our surprise, parent reported non-adherence (31.37%) was higher than Child self-reported non-adherence (25.00%) in this study, however, the difference being not statistically significant. This was different from the findings of (Alsous et al., 2020), (Reed-Knight et al., 2011), but (Ingerski et al., 2010). Furthermore, it revealed that children reported lower scores for unintentional non-adherence and tended to have higher scores for intentional non-adherence, compared with parent-reported results, but the differences between them were not statistically significant. This may be why parents reported a higher proportion of non-adherence than children reported outcomes, children may gained an advantage in intentional non-adherence over the advantage parents gained in unintentional non-adherence. On the other hand, it may also be due to the small sample size, which needs to be increased in the future.

In this study, we found that longer disease duration (3 years and above) and parents with poor knowledge about the disease were independent risk factors for poor medication adherence, while other factors, such as age, family economic level, medication frequency, and treatment with monotherapy or multiple medications did not show a significant effect, similar to and different from previous findings. Kitney et al. (2009) also found that medication non-adherence in children was significantly associated with longer disease duration, and Lim et al. (Tanaka et al., 2021) showed that patients’ knowledge about previously prescribed medications taken was related to good adherence. However, several studies have reached different conclusions. Greenley et al. (2010) showed that children whose regimen involved >1 daily medication administration reported poorer adherence than those whose regimen involved 1 or <1 daily medication administration time, and children on monotherapy reported better adherence than those on multiple medications. LeLeiko et al. (2013); Kitney et al. (2009) found that the medication adherence of children aged ≥15 years was poorer than that of children aged <15 years. In our study, we did not find the same, possibly due to the small sample size. For example, in this study, only eight of the 119 children with IBD were treated with multiple medications, which was not sufficient for a statistically significant analysis, and needed to be re-evaluated with a larger sample size.

In this study, the total score of the parental CCKNOW was only 8.0 (5.0-10.0), which is highly consistent with the findings of (Colombara et al., 2015). It revealed that the parental knowledge about IBD was sub-optimal and still needs to be improved. In this study, we found that parental knowledge about IBD might be a predictor for medication adherence in children with IBD, and the innovation of the current research lies in that we further analyzed the correlation between the different categories of parental knowledge and medication adherence in children with IBD. To our surprise, both drug and complication knowledge categories were significantly associated with medication adherence in children with IBD, which had not been explored in previous studies. Previously, some scholars (Quan et al., 2003; Colombara et al., 2015) also found that the IBD patients appeared to even more lack knowledge about drugs and complications, but did not explored the correlation with medication adherence. In addition, a number of previous studies have shown that providing knowledge about medication and disease to patients effectively increased treatment adherence (Robinson et al., 2001; Tae et al., 2016; Tiao et al., 2017; Lim et al., 2020; Vernon-Roberts et al., 2020). Interventions including parent education seminar (Vernon-Roberts et al., 2020), Pharmacist Counseling (Tiao et al., 2017), self-management training (Robinson et al., 2001) and so on. However, only (Reed-Knight et al., 2011) revealed that neither adolescents’ nor parents’ report of medication knowledge were significantly associated with medication adherence in children with IBD. It might be due to the small sample size and different knowledge assessment tools. Therefore, in future research, we should incorporate knowledge about IBD, especially the drug knowledge and complication knowledge as key components of implementing targeted strategies to improve medication adherence in children with IBD.

The power of health insurance status as a predictor of medication adherence has not been previously studied, but it could be a risk factor for medication adherence. The actual cost of medicines varies depending on the type of health insurance, which may affect medication adherence in children with IBD, particularly when taking expensive medicines. Ediger et al. (2007) had previously reported the medication cost was the most prominent issue, with 25% of patients reporting that cost often made it difficult for them to take their medication regularly. However, health insurance status was not a significant predictor in this study, and was not significantly different between the adherent and non-adherent groups, this could be due to a lack of statistical power as the small sample size. It may also be that oral medication, such as mercaptopurine and mesalazine, are not as expensive as biologics, so the type of health insurance has no obvious impact on patients’ cost and thus on medication adherence. Similarly, (Nahon et al., 2011) found that deprivation was not a predictor of medication non-adherence in IBD patients due to the ease of access medication in France where IBD patients qualify for payment exemption. The above studies were conducted in adults with IBD, data in children are limited. Current researches are more focused on exploring whether non-adherence will lead to increased medical costs (Kane and Shaya, 2008; Higgins et al., 2009; Elkjaer et al., 2010), while relatively limited research reports on whether cost, such as drug prices and health insurance types, are predictors of non-adherence in IBD patients. However, this potential risk factor deserves more time for further investigation with different medicine, such as biologics, in future studies.

## Limitations

This single-center, cross-sectional study was conducted at a children’s hospital. However, the demographics of these patients reflect only the epidemiology of IBD in Chinese children, meaning that the results may not be generalizable to other nationalities. In addition, this study only used a subjective adherence assessment tool with no objective tool for more comprehensive information, which needs to be supplemented in our next step for more exploration. Further, relatively small sample size may not have provided sufficient statistical power to identify some factors; we will expand the sample size in the future. However, the usual rule of thumb for sample size in binary logistic regression of 5–10 cases per variable was met in our study, and we adopted forward stepwise logistic regression analysis. In addition, about one-third of the children had intentional dose omissions, while about one-quarter experienced self-discontinuation behavior in our study, which suggests that it is necessary to analyze the potential causes of children’s intentional non-adherence. In the future, we will perform further studies to address these limitations.

## Conclusion

We report the Chinese version of the MARS-4 as a useful medication adherence tool in children with IBD of China and further describe the prevalence and predictors of medication non-adherence in these populations. Poor medication adherence was common in children with IBD in China, and subjective forgetting to take medication was the main barrier. Disease duration and the level of parental knowledge about IBD were independent predictors of medication non-adherence. Future studies should be carried out to focus on the interventions of disease-related knowledge education, especially drug and complication knowledge education, and medication reminders to promote medication adherence in children with IBD. In addition, identifying the adherence threshold necessary for medication efficacy will be of great value in clinical practice, it will provide a basis for when to initiate interventions.

## Data Availability

The original contributions presented in the study are included in the article/supplementary material, further inquiries can be directed to the corresponding authors.

## References

[B1] Afzali A. Katz S. (2018). Inflammatory bowel disease in the baby to baby boomer: Pediatric and elderly Onset of IBD. Curr. Treat. Options Gastroenterol. 16 (3), 289–305. 10.1007/s11938-018-0188-9 30006766

[B2] Alsous M. M. Hawwa A. F. Imrie C. Szabo A. Alefishat E. Farha R. A. (2020). Adherence to azathioprine/6-mercaptopurine in children and adolescents with inflammatory bowel diseases: A multimethod study. Can. J. Gastroenterol. Hepatol. 2020, 9562192. 10.1155/2020/9562192 32185153 PMC7060881

[B3] Benchimol E. I. Bernstein C. N. Bitton A. Carroll M. W. Singh H. Otley A. R. (2017). Trends in epidemiology of pediatric inflammatory bowel disease in Canada: Distributed network analysis of multiple population-based provincial health administrative databases. Am. J. Gastroenterol. 112 (7), 1120–1134. 10.1038/ajg.2017.97 28417994 PMC5527278

[B4] Benchimol E. I. Fortinsky K. J. Gozdyra P. Van den Heuvel M. Van Limbergen J. Griffiths A. M. (2011). Epidemiology of pediatric inflammatory bowel disease: A systematic review of international trends. Inflamm. Bowel Dis. 17 (1), 423–439. 10.1002/ibd.21349 20564651

[B5] Bermejo F. López-San Román A. Algaba A. Guerra I. Valer P. García-Garzón S. (2010). Factors that modify therapy adherence in patients with inflammatory bowel disease. J. Crohns Colitis 4 (4), 422–426. 10.1016/j.crohns.2010.01.005 21122538

[B6] Bland J. M. Altman D. G. (1986). Statistical methods for assessing agreement between two methods of clinical measurement. Lancet 1 (8476), 307–310. 10.1016/s0140-6736(86)90837-8 2868172

[B7] Collyer H. Eisler I. Woolgar M. (2020). Systematic literature review and meta-analysis of the relationship between adherence, competence and outcome in psychotherapy for children and adolescents. Eur. Child. Adolesc. Psychiatry 29 (4), 417–431. 10.1007/s00787-018-1265-2 30604132 PMC7103576

[B8] Colombara F. Martinato M. Girardin G. Gregori D. (2015). Higher levels of knowledge reduce health care costs in patients with inflammatory bowel disease. Inflamm. Bowel Dis. 21 (3), 615–622. 10.1097/MIB.0000000000000304 25636120

[B9] Eaden J. A. Abrams K. Mayberry J. F. (1999). The crohn's and colitis knowledge score: A test for measuring patient knowledge in inflammatory bowel disease. Am. J. Gastroenterol. 94 (12), 3560–3566. 10.1111/j.1572-0241.1999.01536.x 10606319

[B10] Ediger J. P. Walker J. R. Graff L. Lix L. Clara I. Rawsthorne P. (2007). Predictors of medication adherence in inflammatory bowel disease. Am. J. Gastroenterol. 102 (7), 1417–1426. 10.1111/j.1572-0241.2007.01212.x 17437505

[B11] Elkjaer M. Shuhaibar M. Burisch J. Bailey Y. Scherfig H. Laugesen B. (2010). E-Health empowers patients with ulcerative colitis: A randomised controlled trial of the web-guided 'constant-care' approach. Gut 59 (12), 1652–1661. 10.1136/gut.2010.220160 21071584

[B12] Gray W. N. Denson L. A. Baldassano R. N. Hommel K. A. (2012). Treatment adherence in adolescents with inflammatory bowel disease: The collective impact of barriers to adherence and anxiety/depressive symptoms. J. Pediatr. Psychol. 37 (3), 282–291. 10.1093/jpepsy/jsr092 22080456 PMC3306169

[B13] Greenley R. N. Gumidyala A. P. Nguyen E. Plevinsky J. M. Poulopoulos N. Thomason M. M. (2015). Can you teach a teen New tricks? Problem solving skills training improves oral medication adherence in pediatric patients with inflammatory bowel disease participating in a randomized trial. Inflamm. Bowel Dis. 21 (11), 2649–2657. 10.1097/MIB.0000000000000530 26218142

[B14] Greenley R. N. Kunz J. H. Biank V. Martinez A. Miranda A. Noe J. (2012). Identifying youth nonadherence in clinical settings: Data-based recommendations for children and adolescents with inflammatory bowel disease. Inflamm. Bowel Dis. 18 (7), 1254–1259. 10.1002/ibd.21859 22689633

[B15] Greenley R. N. Stephens M. Doughty A. Raboin T. Kugathasan S. (2010). Barriers to adherence among adolescents with inflammatory bowel disease. Inflamm. Bowel Dis. 16 (1), 36–41. 10.1002/ibd.20988 19434722

[B16] Higgins P. D. Rubin D. T. Kaulback K. Schoenfield P. S. Kane S. V. (2009). Systematic review: Impact of non-adherence to 5-aminosalicylic acid products on the frequency and cost of ulcerative colitis flares. Aliment. Pharmacol. Ther. 29 (3), 247–257. 10.1111/j.1365-2036.2008.03865.x 18945258

[B17] Hommel K. A. Davis C. M. Baldassano R. N. (2009). Objective versus subjective assessment of oral medication adherence in pediatric inflammatory bowel disease. Inflamm. Bowel Dis. 15 (4), 589–593. 10.1002/ibd.20798 18985746 PMC2663377

[B18] Horne R. Parham R. Driscoll R. Robinson A. (2009). Patients' attitudes to medicines and adherence to maintenance treatment in inflammatory bowel disease. Inflamm. Bowel Dis. 15 (6), 837–844. 10.1002/ibd.20846 19107771

[B19] Ingerski L. M. Baldassano R. N. Denson L. A. Hommel K. A. (2010). Barriers to oral medication adherence for adolescents with inflammatory bowel disease. J. Pediatr. Psychol. 35 (6), 683–691. 10.1093/jpepsy/jsp085 19776229 PMC2902844

[B20] Jacobsen R. Møldrup C. Christrup L. Sjøgren P. Hansen O. B. (2009). The Danish version of the medication adherence report scale: Preliminary validation in cancer pain patients. Pain Pract. 9 (1), 1–7. 10.1111/j.1533-2500.2008.00245.x 19019056

[B21] Jeganathan J. Lee C. H. Rahme A. Tiao D. K. Weston C. Dutt S. (2017). Pediatric-to-adult transition and medication adherence in patients with inflammatory bowel disease. Inflamm. Bowel Dis. 23 (7), 1065–1070. 10.1097/MIB.0000000000001114 28498154

[B22] Kane S. Shaya F. (2008). Medication non-adherence is associated with increased medical health care costs. Dig. Dis. Sci. 53 (4), 1020–1024. 10.1007/s10620-007-9968-0 17934828

[B23] Keerthy D. Youk A. Srinath A. I. Malas N. Bujoreanu S. Bousvaros A. (2016). Effect of psychotherapy on health care utilization in children with inflammatory bowel disease and depression. J. Pediatr. Gastroenterol. Nutr. 63 (6), 658–664. 10.1097/MPG.0000000000001207 27035372 PMC5040612

[B24] Kitney L. Turner J. M. Spady D. Malik B. El-Matary W. Persad R. (2009). Predictors of medication adherence in pediatric inflammatory bowel disease patients at the Stollery Children's Hospital. Can. J. gastroenterology = J. Can. de gastroenterology 23 (12), 811–815. 10.1155/2009/536860 PMC2805517 20011733

[B25] LeLeiko N. S. Lobato D. Hagin S. McQuaid E. Seifer R. Kopel S. J. (2013). Rates and predictors of oral medication adherence in pediatric patients with IBD. Inflamm. Bowel Dis. 19 (4), 832–839. 10.1097/MIB.0b013e3182802b57 23446336 PMC5704966

[B26] Lim J. K. Lee Y. J. Park J. H. (2020). Medication-related knowledge and medication adherence in pediatric and adolescent patients with inflammatory bowel disease. J. Korean Med. Sci. 35 (14), e92. 10.3346/jkms.2020.35.e92 32281312 PMC7152532

[B27] Loftus E. V. Jr Guérin A. Yu A. P. Wu E. Q. Yang M. Chao J. (2011). Increased risks of developing anxiety and depression in young patients with Crohn's disease. Am. J. Gastroenterol. 106 (9), 1670–1677. 10.1038/ajg.2011.142 21537359

[B28] Nahon S. Lahmek P. Saas C. Durance C. Olympie A. Lesgourgues B. (2011). Socioeconomic and psychological factors associated with nonadherence to treatment in inflammatory bowel disease patients: Results of the ISSEO survey. Inflamm. Bowel Dis. 17 (6), 1270–1276. 10.1002/ibd.21482 21560190

[B29] Oliva-Hemker M. M. Abadom V. Cuffari C. Thompson R. E. (2007). Nonadherence with thiopurine immunomodulator and mesalamine medications in children with Crohn disease. J. Pediatr. Gastroenterol. Nutr. 44 (2), 180–184. 10.1097/MPG.0b013e31802b320e 17255828

[B30] Pearce C. J. Fleming L. (2018). Adherence to medication in children and adolescents with asthma: Methods for monitoring and intervention. Expert Rev. Clin. Immunol. 14 (12), 1055–1063. 10.1080/1744666X.2018.1532290 30286679

[B31] Piovani D. Danese S. Peyrin-Biroulet L. Bonovas S. (2020). Inflammatory bowel disease: Estimates from the global burden of disease 2017 study. Aliment. Pharmacol. Ther. 51 (2), 261–270. 10.1111/apt.15542 31660629

[B32] Plevinsky J. M. Wojtowicz A. A. Miller S. A. Greenley R. N. (2019). Longitudinal barriers to thiopurine adherence in adolescents with inflammatory bowel diseases. J. Pediatr. Psychol. 44 (1), 52–60. 10.1093/jpepsy/jsy062 30137372

[B33] Quan H. Present J. W. Sutherland L. R. (2003). Evaluation of educational programs in inflammatory bowel disease. Inflamm. Bowel Dis. 9 (6), 356–362. 10.1097/00054725-200311000-00003 14671484

[B34] Reed-Knight B. Lewis J. D. Blount R. L. (2011). Association of disease, adolescent, and family factors with medication adherence in pediatric inflammatory bowel disease. J. Pediatr. Psychol. 3623 (312), 308811–317815. 10.1093/jpepsy/jsq0763710.1155/2009/536860 20798185

[B35] Robinson A. Thompson D. G. Wilkin D. Roberts C. and Northwest Gastrointestinal Research Group (2001). Guided self-management and patient-directed follow-up of ulcerative colitis: A randomised trial. Lancet (London, Engl. 358 (9286), 976–981. 10.1016/S0140-6736(01)06105-0 11583752

[B36] Rosen M. J. Dhawan A. Saeed S. A. (2015). Inflammatory bowel disease in children and adolescents. JAMA Pediatr. 169 (11), 1053–1060. 10.1001/jamapediatrics.2015.1982 26414706 PMC4702263

[B37] Schurman J. V. Cushing C. C. Carpenter E. Christenson K. (2011). Volitional and accidental nonadherence to pediatric inflammatory bowel disease treatment plans: Initial investigation of associations with quality of life and disease activity. J. Pediatr. Psychol. 36 (1), 116–125. 10.1093/jpepsy/jsq046 20498007

[B38] Selinger C. P. Eaden J. Jones D. B. Katelaris P. Chapman G. McDonald C. (2013). Modifiable factors associated with nonadherence to maintenance medication for inflammatory bowel disease. Inflamm. Bowel Dis. 19 (10), 2199–2206. 10.1097/MIB.0b013e31829ed8a6 23899547

[B39] Selinger C. P. Ochieng A. O. George V. Leong R. W. (2019). The accuracy of adherence self-report scales in patients on thiopurines for inflammatory bowel disease: A comparison with drug metabolite levels and medication possession ratios. Inflamm. Bowel Dis. 25 (5), 919–924. 10.1093/ibd/izy309 30265299

[B40] Spekhorst L. M. Hummel T. Z. Benninga M. A. van Rheenen P. F. Kindermann A. (2016). Adherence to oral maintenance treatment in adolescents with inflammatory bowel disease. J. Pediatr. Gastroenterol. Nutr. 62 (2), 264–270. 10.1097/MPG.0000000000000924 26230905

[B41] Tae C. H. Jung S. A. Moon H. S. Seo J. A. Song H. K. Moon C. M. (2016). Importance of patients' knowledge of their prescribed medication in improving treatment adherence in inflammatory bowel disease. J. Clin. Gastroenterol. 50 (2), 157–162. 10.1097/MCG.0000000000000431 26501880

[B42] Tanaka M. Kawakami A. Maeda S. Kunisaki R. Morisky D. E. (2021). Validity and reliability of the Japanese version of the morisky medication adherence scale-8 in patients with ulcerative colitis. Gastroenterol. Nurs. 44 (1), 31–38. 10.1097/SGA.0000000000000533 33351521

[B43] Tiao D. K. Chan W. Jeganathan J. Chan J. T. Perry J. Selinger C. P. (2017). Inflammatory bowel disease pharmacist adherence counseling improves medication adherence in crohn's disease and ulcerative colitis. Inflamm. Bowel Dis. 23 (8), 1257–1261. 10.1097/MIB.0000000000001194 28719539

[B44] Vernon-Roberts A. Gearry R. B. Day A. S. (2020). Assessment of knowledge levels following an education program for parents of children with inflammatory bowel disease. Front. Pediatr. 8, 475. 10.3389/fped.2020.00475 32903635 PMC7438864

[B45] Wu Y. P. Pai A. L. Gray W. N. Denson L. A. Hommel K. A. (2013). Development and reliability of a correction factor for family-reported medication adherence: Pediatric inflammatory bowel disease as an exemplar. J. Pediatr. Psychol. 38 (8), 893–901. 10.1093/jpepsy/jst043 23804406 PMC3859234

[B46] Xiao B. Laroui H. Viennois E. Ayyadurai S. Charania M. A. Zhang Y. (2014). Nanoparticles with surface antibody against CD98 and carrying CD98 small interfering RNA reduce colitis in mice. Gastroenterology 146 (5), 1289–1300. e3019. 10.1053/j.gastro.2014.01.056 24503126 PMC3992175

[B47] Zhu Y. Zheng L. Bian Q. G. Ding X. F. (2012). Study on reliability and validity of knowledge questionnaire of Chinese version of Crohn’s disease and colitis. Chin. Nurs. Res. 27 (30), 3449–3451. 10.3969/j.issn.1009-6493.2013.30.065

